# Development of a human milk concentrate with human milk lyophilizate for feeding very low birth weight preterm infants: A preclinical experimental study

**DOI:** 10.1371/journal.pone.0210999

**Published:** 2019-02-20

**Authors:** Mariana M. Oliveira, Davi C. Aragon, Vanessa S. Bomfim, Tânia M. B. Trevilato, Larissa G. Alves, Anália R. Heck, Francisco E. Martinez, José S. Camelo

**Affiliations:** 1 Department of Pediatrics, Children´s Hospital, Ribeirão Preto Medical School, University of São Paulo, Ribeirão Preto, São Paulo, Brazil; 2 Laboratory of Pediatrics, Clinics Hospital of Ribeirão Preto, Ribeirão Preto Medical School, University of São Paulo, Ribeirão Preto, São Paulo, Brazil; 3 Human Milk Bank, Clinics Hospital of Ribeirão Preto, Ribeirão Preto, São Paulo, Brazil; 4 Department of Pediatrics, Neonatology, Children´s Hospital, Ribeirão Preto Medical School, University of São Paulo, Ribeirão Preto, São Paulo, Brazil; University of Illinois, UNITED STATES

## Abstract

Breast milk is considered the gold standard nutritional resource for very low birth weight (VLBW) infants in terms of nutrients and protective factors. If mother's milk is not available, the second choice is donated and fortified human milk (HM) from the Human Milk Bank (HMB). This study hypothesized that HM could be lyophilized and used as an additive to increase the levels of macronutrients and micronutrients available to VLBW infants. This study aimed to constitute a lyophilized HM concentrate and determine the osmolality and the concentration of macronutrients and micronutrients in HM samples at “baseline” and in “HM concentrates”, analyzed immediately (HMCI), and after 3 (HMC3m) and 6 (HMC6m) months of freezing. Osmolality was verified using the freezing point osmometric method. Macronutrient quantification was performed using the MIRIS Human Milk Analyzer. Micronutrients were determined by Flame Atomic Absorption Spectrophotometry and by the automated colorimetric method. Bayesian linear mixed effect models were adjusted using OpenBUGS to estimate mean differences and 95% credibility intervals (CrI) of osmolality and of macro- and micronutrients between the types of HM samples. A comparison of dosage values showed a significant increase between HM baseline and HMCI, HMC3m, and HMC6m. Comparing HM baseline and HMCI highlighted the increase in energy content and the concentration of carbohydrates and total lipids. The Ca and P contents increased and the levels of energy, total lipids, and Cu were reduced in HMC3m compared to HMCI. Ca, Mg, K, Zn, and P increased and the levels of energy, total lipids, and Cu were reduced in HMC6m, compared to HMCI. The present study confirms the possibility of formulation and utilization of the immediate concentrate. Partial stability of HM concentrates generated from freeze-drying of donated milk do not recommend storage.

## 1. Introduction

Despite considerable advances, the optimal nutritional support for very low birth weight infants (<1500 g) has not yet been found. The current aims of neonatal care include promoting survival as well as mimicking fetal growth and proper neurodevelopment of these extremely immature infants. Providing for the nutritional needs of very low birth weight (VLBW) infants is a challenge since the preterm stage has a higher demand for energy, protein, and other nutrients than other development stages, as infants have low stores of key nutrients such as iron, zinc, and calcium. An adequate supply of energy and protein during the first week of life is associated with improved growth and development up to the post-conceptional age of 2 years. In addition, the physiological immaturity, diseases, and general stress of prematurity means that these infants require even more optimal nutritional support [1–6].

Breast milk is considered the gold standard nutritional resource due to the provision of nutrients and protective factors. Some advantages conferred to VLBW infants improved long-term outcomes of neurodevelopment, immune defenses, development of the microbiota and the gastrointestinal system, and reduced incidence of necrotizing enterocolitis (NEC) and sepsis [7–10]. In addition, early enteral nutrition with an exclusively human milk (HM) diet has proven to be an effective nutritional support strategy for VLBW infants because of its association with lower incidence of NEC, prevention of neonatal infant mortality, and reduction of time and cost of hospital admission with each preterm infant [11–13].

Current guidelines suggest that all preterm infants should receive HM and that the first choice should be the mother's own milk. Nonetheless, HM should be fortified to ensure optimal nutrient intake. If the own mother's milk is not available, the second choice is donated and fortified HM from the Human Milk Bank (HMB). The nutritional content of donated HM by itself may not satisfy the special nutritional needs of VLBW infants, especially for proteins, micronutrients, and energy, thereby jeopardizing the growth and development of these preterm infants [14–17]. A recent study reported that the adequacy of the quantity and quality of protein intake by VLBW infants influences the rate and relative quality of weight gain [18]. Moreover, an inadequate supply of the micronutrients calcium, phosphorus, and zinc can also culminate in deficient growth and development since these nutrients are responsible for maturation and for the functionality of several enzymatic systems [19–20]. Faced with the impossibility of increasing the volume of HM and thus the absorption of nutrients [21], the strategy to adapt this milk to the nutritional needs of VLBW infants is through the fortification of HM with commercial additives to be added to the mother's own milk (raw or pasteurized) or donated HM. However, widely used bovine milk protein-based products, despite providing growth and weight gain to preterm infants, partially alter the immunological quality of HM, increase osmolarity and risk of sensitization by heterologous protein, as well as the occurrence of NEC [22–25]. More recently, human milk-based additives have been formulated and studies show the advantages of their use, such as improved morbidity and mortality rates, less incidence of NEC, decreased hospital stays, and improvement of infant growth and weight gain. Nevertheless, the cost of the product and the ethical questions related to its commercialization make it difficult to use it in neonatal intensive care units (ICUs) [26–29]. Thus, considering the recent scientific breakthroughs, the hypothesis of this study is that HM voluntarily donated to the HMB may be lyophilized, in a simple and effortless way, and used as an additive of HM to increase the levels of nutrients. The aims of this study were to constitute a concentrate with freeze-dried HM and determine the osmolality and the concentration of macronutrients and micronutrients in HM samples immediately and after 3 and 6 months in storage to evaluate the increase in concentration of nutrients followed by nutritional stability of the product. This study reports an innovative and simplified proposal of nutritional support for VLBW infants with an exclusive HM diet to be implemented in the routine of the Human Milk Bank Network of Brazil (HMB Network Brazil) to ensure the nutrition of preterm infants and as an alternative to use of the artificial additives currently being used.

## 2. Materials and methods

This research project was carried out through a partnership with the HMB and the Laboratory of Metals and Rare Diseases–Pediatrics, at the Clinics Hospital of Ribeirão Preto, Ribeirão Preto Medical School, University of São Paulo (USP). The milk used in this study came from donations of surplus production of HM, that is, without compromising the child's own feeding. Since this study was carried out with human biological material, it was submitted to and approved by the Human Research Ethics Committee of the Clinics Hospital, Ribeirão Preto Medical School—USP (HREC Report No. 738.080). The donors were informed about the nature of the study and those who were willing to participate in the project signed a free and informed consent form; donors also underwent clinical and serological screening.

### Material collection, processing, and quality control

The donors with a lactation period greater than 15 days were given instructions about massaging and milking their breasts, and about how to withdraw the milk into a sterile, inert glass bottle provided by the HMB. All the samples passed through the selection and classification processes recommended by the HMB Network Brazil (available at: http://www.redeblh.fiocruz.br). The selection process included packaging conditions, presence of dirt, color, off-flavor parameters, and Dornic acidity. The classification process included the verification of the lactation period, Dornic acidity, and energetic content—the crematocrit [30–33]. Considering a value of 0.36 for the standard deviation with respect to the expected average of protein concentration (2.20 g/dL) and an absolute error value of 0.1, with a confidence level of 95%, 50 samples were obtained. Additional data were collected to characterize the donors: age, weight, height, Body Mass Index (BMI), and gestational age. Inclusion criteria were surplus mature HM with a Dornic acidity value of up to 8°D.

### Obtaining the HM concentrate

For lyophilization, 50 mL of donated HM was transferred to an inert, sterile glass container and frozen (-20°C for 24 hours). After this period, the frozen sample was placed in the vacuum chamber of a bench lyophilizer (Lyophilizer L108, LioTop, São Carlos—SP—Brazil). After 72 hours, the lyophilized sample was transferred from the lyophilizer to a cold chain, to be reconstituted with HM for use. The concentrate with the HM lyophilizate in the immediate period (HMCI) was composed from samples that were withdrawn from the lyophilizer and reconstituted with 75 mL of the donor's own HM baseline. These concentrates together with the HM baseline were passed through the processes of pasteurization and microbiological quality control. HM baseline and HMCI were pasteurized at 62.5°C for 30 minutes after a preheating period [34]. After 30 minutes of thermal treatment that is lethal to pathogenic bacteria, the vials were withdrawn from the bath and cooled until the HM reached a temperature ≤ 5°C. For the microbiological quality control check, the pasteurized HM baseline and HMCI samples were screened for total coliforms using bright green bile broth (50g/L; 5% w/v) contained within Durham tubes. The concentrate with the HM lyophilizate in the immediate period was subdivided in collection tubes and stored (-20°C) for 3 (HMC3m) and 6 (HMC6) months, to evaluate nutritional stability. Two hundred types of HM samples (50 of each type: HM baseline, HMCI, HMC3m, and HMC6m) were analyzed for osmolality, macronutrients, and micronutrients. Osmolality verification was performed using the freezing point osmometric method in the PZL-1000 Microprocessed Osmometer (PZL).

### Macronutrients

In the HMB, the 200 types of HM samples were homogenized using a Sonicator MIRIS (Miris, Uppsala, Sweden), and the macronutrient content (protein, carbohydrate, total lipids, total solids and true protein) was quantified in 2 mL aliquots of each sample type by using the MIRIS Human Milk Analyzer (Miris, Uppsala, Sweden). The MIRIS Human Milk Analyzer performs precise and accurate analyses based on infrared transmission spectroscopy. Sample types HMC3m and HMC6m were previously defrosted in a water bath at 37°C.

### Micronutrients

After the pasteurization process, aliquots of each type of HM sample were immediately separated in collection tubes, frozen (-20°C for 24 hours), and sent to the Laboratory of Metals and Rare Diseases for immediate analysis of micronutrients and also 3 and 6 months post-freezing. The micronutrients Calcium (Ca), Magnesium (Mg), Sodium (Na), Potassium (K), Copper (Cu), Zinc (Zn) and Phosphorus (P) were determined “in natura”, after defrosting samples in a water bath at 37°C followed by homogenization of the material by ultrasound using Sonicator MIRIS. Proper dilutions were made within the concentration ranges of the calibration curves for readings in the Flame Atomic Absorption Spectrophotometry (EAA 55B VARIAN), in which each element uses a hollow-cathode lamp and specific wavelength, acetylene gas and compressed air. Phosphorus was dosed in the Metrolab equipment with a kit from Wiener Lab., Ref 1382321 by the automated colorimetric method.

### Statistical analysis

The exploratory analysis of osmolality dosages and content of macronutrients and micronutrients was carried out through the mean values (standard deviation) and boxplots (S1 Fig). Bayesian linear mixed effects models were adjusted using OpenBUGS to estimate the mean difference and 95% credibility intervals when comparing the dosages of osmolality and of the macronutrients and micronutrients between the types of HM sample: HM baseline, HMCI, HMC3m, and HMC6m.

## 3. Results

Fifty HM donors participated in the study. After the selection and classification of the samples according to the inclusion criteria, the mean values (standard deviation) of the Dornic acid values were 4.34°D (1.59). The characteristics of the HM donors are summarized in Table 1, in which the mean (standard deviation) age, weight, height, BMI, and gestational age was, respectively, 30.45 (6.05) years, 67.52 (11.92) kg, 1.63 (0.59) m, 25.2 (4.39) kg/m^2^, and 38.43 (2.27) weeks.

**Table 1:** Characteristics of the HM donors.

Mothers (n = 50)	Mean	Standard Deviation	Minimum	Maximum
Age (years)	30.45	6.05	17	44
Weight (kg)	67.52	11.92	47	95
Height (m)	1.63	0.59	1.52	1.77
BMI (kg/m^2^)	25.2	4.39	18.1	38.1
Gestational age (weeks)	38.43	2.27	27	42

The descriptive dosage results of the mean (standard deviation) of osmolality and content of macronutrients and micronutrients in types of samples HM baseline, HMCI, HMC3m, and HMC6m presented in Table 2. An increase in the levels of the evaluated nutrients was observed in the concentrates of HMCI, HMC3m, and HMC6m, compared to the HM baseline.

**Table 2:** Descriptive statistics of dosage of osmolality and content of macronutrients and micronutrients in HM baseline and the concentrates HMCI, HMC3m, and HMC6m.

Dosages (units)	HM baseline	HMCI	HMC3m	HMC6m
Energy (Kcal/100 mL)	56.30 (10.51)	79.96 (13.75)	76.98 (13.91)	77.30 (13.78)
Protein (g/100 mL)	0.90 (0.49)	1.48 (0.58)	1.39 (0.61)	1.47 (0.54)
Carbohydrate (g/100 mL)	7.08 (0.67)	9.18 (0.68)	9.21 (0.63)	9.18 (0.64)
Total lipids (g/100 mL)	2.59 (1.08)	4.03 (1.44)	3.68 (1.34)	3.69 (1.35)
Total solids (g/100 mL)	10.76 (1.31)	14.77 (1.72)	14.48 (1.78)	14.53 (1.71)
True protein (g/100 mL)	0.75 (0.40)	1.20 (0.48)	1.13 (0.49)	1.19 (0.43)
Osmolality (mOsm/Kg H_2_0)	289.48 (43.64)	452.12 (59.79)	456.16 (56.58)	458.14 (55.67)
Calcium (mg/100 mL)	23.24 (4.70)	36.52 (7.18)	38.67 (6.25)	40.63 (6.02)
Magnesium (mEq/L)	2.14 (0.46)	3.38 (0.65)	3.52 (0.83)	3.73 (0.90)
Sodium (mg/L)	135.04 (98.03)	222.52 (169.03)	244.87 (162.80)	233.19 (141.53)
Potassium (mg/L)	601.38 (147.98)	1013.85 (269.45)	1015.04 (222.82)	1152.31 (234.18)
Copper (μg/100 mL)	33.68 (14.60)	48.30 (19.99)	41.16 (15.12)	39.28 (13.71)
Zinc (μg/100 mL)	149.10 (128.15)	203.89 (126.20)	224.46 (152.50)	251.70 (158.76)
Phosphorus (mg/100 mL)	14.63 (6.09)	18.47 (5.97)	20.36 (7.52)	20.50 (7.35)

HM baseline: Human milk baseline; HMCI: HM concentrated for immediate analysis; HMC3m: HM concentrate for analysis after 3 months of storage; HMC6m: HM concentrate for analysis after 6 months of storage; Mean (standard deviation).

The comparative values of dosages of osmolality and of macronutrients and micronutrients in the samples of the types (Table 3) show that there was a significant increase in all the parameters in HMCI, HMC3m, and HMC6m, compared to HM baseline. This highlights the increase in energy content (-23.680; 95% CrI [-25.910; -21.450]) and the concentration of carbohydrates (-2.095; 95% CrI [-2.286; -1.912]) and total lipids (-1.437; 95% CrI [-1.678; -1.193]). In HMC3m samples, a significant increase was observed in the levels of element Ca (-2.139; 95% CrI [-3.356; -0.971]) and P (-1.882; 95% CrI [-3.618; -0.209]), but a slight reduction in the levels of energy (2.988; 95% CrI [0.689; 5.193]) and total lipids (0.342; 95% CrI [0.099; 0.591]), while the osmolality and other nutrients remained stable, except for Cu (7.154; 95% CrI [3.840; 10.330]), compared to HMCI samples. In HMC6m samples, a significant increase was observed in the levels of Ca (-4.103; 95% CrI [-5.325; -2.904]), Mg (-0.357; 95% CrI [-0.515; -0.201]), K (-131.400; 95% CrI [-193.400; -70.690]), Zn (-47.290; 95% CrI [-72.040; -23.100]), and P (-2.022; 95% CrI [-3.738; -0.355]), but a slight reduction in the levels of energy (2.672; 95% CrI [0.417; 4.878]) and total lipids (0.335; 95% CrI [0.095; 0.579]), while the osmolality and other nutrients remained stable, except for Cu (9.040; 95% CrI [5.782; 12.203]), compared to HMCI samples. Finally, in HMC6m samples, stored for 6 months, a significant increase in the levels of Ca (-1.965; 95% CrI [-3.203; -0.770]), Mg (-0.216; 95% CrI [-0.377; -0.061]), K (-130.400; 95% CrI [-193.400; -69.810]), and Zn (-26.980; 95% CrI [-51.910; -2.026]) was observed, compared to HMC3m samples. The mean Cu levels presented a different pattern in relation to the rest. A significant decrease in Cu content in the comparison between HMCI and HMC3m and HMCI and HMC6m shows that the storage period reduced the content of this micronutrient.

**Table 3:** Mean difference of dosages of osmolality and content of macronutrients and micronutrients analyzed between HM baseline and concentrates HMCI, HMC3m, and HMC6m.

Variable	Comparisons	Mean difference	95% CrI lower limit	95% CrI upper limit
Energy (kcal/100 mL)	HM baseline—HMCI	**-23.680**	**-25.910**	**-21.450**
	HM baseline—HMC3m	**-20.690**	**-22.930**	**-18.410**
	HM baseline—HMC6m	**-21.000**	**-23.240**	**-18.750**
	HMCI—HMC3m	**2.988**	**0.689**	**5.193**
	HMCI—HMC6m	**2.672**	**0.417**	**4.878**
	HMC3m - HMC6m	-0.316	-2.598	1.951
Proteins (g/100 mL)	HM baseline—HMCI	**-0.576**	**-0.668**	**-0.482**
	HM baseline—HMC3m	**-0.490**	**-0.584**	**-0.394**
	HM baseline—HMC6m	**-0.566**	**-0.660**	**-0.472**
	HMCI—HMC3m	0.086	-0.010	0.179
	HMCI—HMC6m	0.010	-0.086	0.105
	HMC3m - HMC6m	-0.076	-0.174	0.018
Carbohydrates (g/100 mL)	HM baseline—HMCI	**-2.095**	**-2.286**	**-1.912**
	HM baseline—HMC3m	**-2.126**	**-2.307**	**-1.934**
	HM baseline—HMC6m	**-2.099**	**-2.286**	**-1.912**
	HMCI—HMC3m	-0.030	-0.220	0.151
	HMCI—HMC6m	-0.004	-0.189	0.176
	HMC3m - HMC6m	0.026	-0.161	0.209
Total lipids (g/100 mL)	HM baseline—HMCI	**-1.437**	**-1.678**	**-1.193**
	HM baseline—HMC3m	**-1.095**	**-1.340**	**-0.845**
	HM baseline—HMC6m	**-1.102**	**-1.342**	**-0.863**
	HMCI—HMC3m	**0.342**	**0.099**	**0.591**
	HMCI—HMC6m	**0.335**	**0.095**	**0.579**
	HMC3m - HMC6m	-0.006	-0.248	0.233
Total solids (g/100 mL)	HM baseline—HMCI	**-4.004**	**-4.307**	**-3.703**
	HM baseline—HMC3m	**-3.715**	**-4.020**	**-3.406**
	HM baseline—HMC6m	**-3.769**	**-4.072**	**-3.463**
	HMCI—HMC3m	0.289	-0.023	0.588
	HMCI—HMC6m	0.236	-0.070	0.535
	HMC3m - HMC6m	-0.053	-0.363	0.254
True protein (g/100 mL)	HM baseline—HMCI	**-0.453**	**-0.531**	**-0.375**
	HM baseline—HMC3m	**-0.380**	**-0.458**	**-0.301**
	HM baseline—HMC6m	**-0.444**	**-0.522**	**-0.366**
	HMCI—HMC3m	0.072	-0.008	0.149
	HMCI—HMC6m	0.008	-0.070	0.085
	HMC3m - HMC6m	-0.064	-0.143	0.015
Osmolality (mOsm/Kg H_2_0)	HM baseline—HMCI	**-162.200**	**-177.900**	**-146.600**
	HM baseline—HMC3m	**-166.200**	**-182.000**	**-150.300**
	HM baseline—HMC6m	**-168.100**	**-183.700**	**-152.300**
	HMCI—HMC3m	-3.975	-20.040	11.420
	HMCI—HMC6m	-5.920	-21.630	9.556
	HMC3m - HMC6m	-1.945	-17.880	13.980
Calcium (mg/100 mL)	HM baseline—HMCI	**-13.290**	**-14.460**	**-12.100**
	HM baseline—HMC3m	**-15.420**	**-16.620**	**-14.210**
	HM baseline—HMC6m	**-17.390**	**-18.580**	**-16.200**
	HMCI—HMC3m	**-2.139**	**-3.356**	**-0.971**
	HMCI—HMC6m	**-4.103**	**-5.325**	**-2.904**
	HMC3m - HMC6m	**-1.965**	**-3.203**	**-0.770**
Magnesium (mEq/L)	HM baseline—HMCI	**-1.234**	**-1.386**	**-1.080**
	HM baseline—HMC3m	**-1.376**	**-1.532**	**-1.219**
	HM baseline—HMC6m	**-1.591**	**-1.747**	**-1.437**
	HMCI—HMC3m	-0.141	-0.298	0.010
	HMCI—HMC6m	**-0.357**	**-0.515**	**-0.201**
	HMC3m - HMC6m	**-0.216**	**-0.377**	**-0.061**
Sodium (mg/L)	HM baseline—HMCI	**-86.600**	**-113.500**	**-58.860**
	HM baseline—HMC3m	**-108.600**	**-136.600**	**-80.480**
	HM baseline—HMC6m	**-97.060**	**-124.900**	**-69.270**
	HMCI—HMC3m	-22.020	-50.450	5.252
	HMCI—HMC6m	-10.460	-38.880	17.470
	HMC3m - HMC6m	11.570	-17.490	39.240
Potassium (mg/L)	HM baseline—HMCI	**-392.100**	**-451.100**	**-331.600**
	HM baseline—HMC3m	**-393.000**	**-453.600**	**-330.500**
	HM baseline—HMC6m	**-523.400**	**-584.100**	**-462.800**
	HMCI—HMC3m	-0.929	-62.620	58.190
	HMCI—HMC6m	**-131.400**	**-193.500**	**-70.690**
	HMC3m - HMC6m	**-130.400**	**-193.400**	**-69.810**
Copper (μg/100 mL)	HM baseline—HMCI	**-14.950**	**-18.200**	**-11.740**
	HM baseline—HMC3m	**-7.799**	**-11.040**	**-4.511**
	HM baseline—HMC6m	**-5.914**	**-9.141**	**-2.656**
	HMCI—HMC3m	**7.154**	**3.840**	**10.330**
	HMCI—HMC6m	**9.040**	**5.782**	**12.230**
	HMC3m - HMC6m	1.885	-1.388	5.166
Zinc (μg/100 mL)	HM baseline—HMCI	**-54.530**	**-79.040**	**-30.160**
	HM baseline—HMC3m	**-74.840**	**-99.420**	**-49.910**
	HM baseline—HMC6m	**-101.800**	**-126.400**	**-77.040**
	HMCI—HMC3m	-20.320	-45.420	3.872
	HMCI—HMC6m	**-47.290**	**-72.040**	**-23.100**
	HMC3m - HMC6m	**-26.980**	**-51.910**	**-2.026**
Phosphorus (mg/100 mL)	HM baseline—HMCI	**-3.859**	**-5.557**	**-2.176**
	HM baseline—HMC3m	**-5.741**	**-7.437**	**-4.020**
	HM baseline—HMC6m	**-5.881**	**-7.575**	**-4.175**
	HMCI—HMC3m	**-1.882**	**-3.618**	**-0.209**
	HMCI—HMC6m	**-2.022**	**-3.738**	**-0.355**
	HMC3m - HMC6m	-0.140	-1.865	1.570

HM baseline: Human milk baseline; HMCI: HM concentrated for immediate analysis; HMC3m: HM concentrate for analysis after 3 months of storage; HMC6m: HM concentrate for analysis after 6 months of storage. 95% CrI: Credibility interval 95%.

## 4. Discussion

Considering the current need for the fortification of HM used in the nutritional support of VLBW infants, this study presents an innovative possibility of formulating a concentrated HM through a simplified method of direct lyophilization of milk donated to HMB. Thus, the merit of this study was to demonstrate the possibility of formulating a product with high nutritional quality components with low cost of production and without ethical bias.

Several authors emphasize the necessary addition of fortifiers to banked human milk in order to increase the nutrient content and thus to be able to meet the special nutritional needs of VLBW infants [2–4]. In the neonatal clinical practice of most public hospitals in Brazil, bovine milk protein-based products such as FM-85 (Nestlé) and Enfamil HMF (Mead Johnson) are used as fortifiers. However, studies report that such products delay gastric emptying and expose preterm infants to the risk of sensitization by heterologous protein and the occurrence of NEC [35–38].

The present study introduces an innovative proposal for the formulation of a human milk-base additive through a simplified direct freeze-drying method followed by a single pasteurization process, which minimizes the risk of contamination and nutrient loss. A similar study carried out in Brazil reports on the development of two human milk-based additives (liquid and powdered) from fat extraction, evaporation, lactose reduction, and lyophilization methods [37]. That same research group has recently improved the formulation method of the powder additive by minimizing the risks of the elaboration and handling processes of HM by simplifying the method to lactose reduction and lyophilization alone. The partial removal of lactose is justified by the control of osmolality of HM strengthened with the powdered additive, which increases tolerance by preterm infants, minimizing the risk of NEC [39–40]. In the present study, the proposed simplified method consists of lyophilization without lactose reduction, and it is important to highlight that the concentrated HM after the addition of the lyophilizate maintained an acceptable osmolality according to the values tolerated by VLBW infants.

The control of osmolality and the physical-chemical and microbiological qualities of HM is essential for its safe provision to VLBW infants. Recent studies show that the procedures carried out in this study, which were established as a protocol by the HMB Network in Brazil, do not change the osmolality or the levels of macronutrients and micronutrients in the milk [31,34,41]. In terms of the lyophilization process, studies report that lyophilizing HM before the freezing process allows for a better preservation of the nutritional properties of milk [42–43]. In this study, the presented values of osmolality and the levels of macronutrients and micronutrients refer to the moment post-lyophilization and pasteurization; therefore, they reflect the final composition of concentrated HM.

The concentrated milk produced in this study is a safe and viable nutritional support alternative since it presents acceptable osmolality and meets the nutritional needs of VLBW infants related to the recommended enteral intake of macronutrients and micronutrients, with few exceptions, and may allow for a reduction in the use of bovine milk protein-based additives. Consensus-based evidence regarding the optimization of nutritional support of VLBW infant reports that the first choice of enteral feeding is own mother's milk fortified with balanced osmolality to ensure safe supply of additional nutrients [4,15]. Studies show that osmolality of a preterm mother's milk is similar to our HM baseline, mature milk donated by "term" mothers in the later stage of lactation, and this allows our lyophilizate to be used as an additive to the milk of the VLBW infant's own mother, thus generating a concentrate with acceptable osmolality [22,40].

Regarding the recommendations of macronutrients and micronutrients expressed per 100 kcal of HMCI presented in S1 Table, the mean and confidence interval (CI) of protein (1.85g; 90% CI [1.68–2.02]), Ca (45.70mg; 90% CI [43.56–47.81]), Mg (5.13mg; 90% CI [4.89–5.36]), Na (27.84mg; 90% CI [22.84–32.83)], Cu (60.42μg; 90% CI [54.51–66.33]), Zn (0.25mg; 90% CI [0.22–0.29)], and P (23.10mg; 90% CI [21.34–24.87]) content partially meet the recommendations of enteral intakes established by the Life Sciences Research Office (LRSO 2002) and the European Society of Pediatric Gastroenterology, Hepatology, and Nutrition (ESPGHAN 2010) for preterm infants. It is important to emphasize that the content of carbohydrate (11.48g; 90% CI [11.28–11.68]), total lipids (5.04g; 90% CI [4.61–5.46]), and K (126.83mg; 90% CI [118.87–134.79]), expressed per 100 kcal of HMCI (S1 Table) fully meet the established recommendations cited above [44–45].

The evolution of neonatal nutritional protocols over the years shows an improvement in the intake of energy and macronutrients through HM and, consequently, better neonatal growth and neurodevelopment [46–47]. Thus, given the updated literature cited and the adequacy of our concentrate on the recommendations of enteral intakes of LRSO 2002 and ESPGHAN 2010, the protein content of our concentrate could raise concerns. However, studies suggest that the quality of human milk protein and the adequate balance of energy and protein intake are associated advantages in rate and quality of growth as well as better clinical outcomes for VLBW infants [18,48–51]. Thus, we believe that our HM concentrate allows an optimization of the nutritional support of premature babies, as it guarantees the supply of high quality bioactive proteins and adequate content of carbohydrates, lipids and K.

The contents of Ca, Mg, Na, Cu, Zn, and P in the concentrated HM sample types also deserve special attention. According to the recommendations of LRSO 2002 and ESPGHAN 2010, these elements may merit isolated supplementation [44–45]. In addition, adequate early intake of protein and energy in the first week of life of VLBW infants improves the homeostasis of the electrolytes in question [52]. Preterm infants require high amounts of Ca and P elements and, due to low skeletal storages, are at increased risk of nutritional disorders such as growth and developmental deficits, hypophosphatemia, osteopenia in prematurity, and metabolic bone disease [53–54].

The essential micronutrients Mg, Zn and Cu are also related to child growth and development as well as immune function [55–57]. A retrospective study confirmed the association between serum levels of Cu and Zn, gestational age and anthropometric parameters of body weight, and body length and head circumference at birth in preterm infants [57]. Watson et al. warned that the most common cause of Zn deficiency is dietary because of the low micronutrient supply that can be generated by the intake of inadequate milk volumes and non-fortified HM [58]. In addition, Cu deficiency is also recurrent in VLBW infants and can cause anemia, neutropenia, failure to thrive, psychomotor retardation, and bone abnormalities [59]. In addition, it is important to note that chronic depletion of Na also negatively affects weight gain and the growth of preterm infants, especially VLBW infants, since they present higher losses of Na and thus require supplementation [60–61]. Therefore, considering the decision to use the HM concentrate produced in this study as a nutritional support strategy for VLBW infants, it will be necessary to monitor and eventually supplement as needed the serum levels of Ca, Mg, Na, Cu, Zn, and P.

The content of macronutrients and micronutrients as well as the osmolality of our concentrated HM has similarities to some currently marketed breast milk fortifiers. The human milk-based additive Prolact +4 H2MF (Prolacta Bioscience) when added to 80 mL of preterm mother's milk reaches an energy content of 82 kcal per 100 mL, according to the information provided by the manufacturer, which is similar to our HMCI as shown in Table 2. However, while our product is able to provide higher amounts of carbohydrates and total lipids per 100 mL (Table 2), Prolacta +4 H2MF provides greater amounts of protein (2.3 g), Na (57 mg), Ca (123 mg), Zn (0.97 mg), and P (64 mg). Despite this, a recent study warned that HM fortified with human milk-based additive does not meet the nutritional recommendations for VLBW infants established by the American Academy of Pediatrics Committee of Nutrition because the supply of protein and vitamins is insufficient when it reaches the recommended caloric intake of 130 kcal/kg [62].

In Brazilian public hospitals, one of the most used HM fortifiers is FM-85 (Nestlé). According to the manufacturer's information, such a bovine milk protein-based additives when added to the HM (100 mL) of the premature mother provides: 85 kcal, 2.5 g of protein, 10.3 g of carbohydrates, 4.02 g of total lipids, 100 mg of Ca, and 59 mg of P. Comparing this nutritional information with the composition of our HMCI (Table 2), we observed similar levels of energy content, protein, and carbohydrates. However, the content of Ca and P present in 100 mL of HMCI is lower than that offered by HM fortified with FM-85 (Nestlé) [22].

Some authors suggest that the use of the FM-85 additive (Nestlé) improves the bone mineralization of VLBW infants, although others report the negative effects of its use [35,36,63]. Sullivan et al. found that the nutritional support of VLBW infants using human milk-based fortifier was associated with lower occurrence of NEC or death when compared to the use of bovine milk protein-based additives [35]. This fact is justified by LH allowing the maternal transfer of adaptive immune defenses, especially secretory IgA, which presents in higher concentration in the maternal milk of mothers of preterm infants [64].

The preservation of nutritional and immunological components as well as the safety of HM processed in HMB is a concern addressed in current literature reviews, as studies and proposals to improve milk manipulation protocols are constantly being generated [32,65]. A study conducted by Ahrabi et al. showed that the process of refrigeration at +4°C for 72 hours followed by frozen storage at -20°C for up to 9 months applied to freshly expressed HM was associated with decreased pH and bacterial counts without affecting the content of total protein, fat, lactoferrin, secretory IgA, and osmolarity in the samples [66]. However, Sousa et al. proved that pasteurization of HM colostrum caused a reduction of 20, 51, and 23% in IgA, IgM, and IgG concentrations, respectively [67]. A literature review concluded a similar reduction of IgA, IgM, and IgG concentrations in HM after holder pasteurization; however, the authors warn that clinical practice demonstrates that many beneficial properties remain after pasteurization, which strongly justifies the use of HM processed in HMB for feeding preterm infants [34]. Therefore, our proposal for the nutritional support of VLBW infants with concentrated HM is also strengthened.

The present study generated a new hypothesis which will be elucidated in future studies: the possibility of adding lyophilized HM directly into the mother's own raw milk to produce an improved concentrated milk. A systematic review presents the differences between nutrient contents according to gestational stage (preterm versus term) and lactation time, emphasizing that the colostrum of mothers of preterm infants presented a higher protein mean when compared with mature HM [68]. Thus, it is likely that our lyophilized milk (50 mL matured donated milk) when added to a VLBW infant’s mother's own raw milk will produce a concentrated HM with a higher protein content and adequate osmolality that can meet the nutritional needs of preterm infants [37,44,68]. Other authors reinforce the initiative to use the mother's own raw milk during the hospitalization period of preterm infants. Dritsakou et al. suggested that feeding VLBW infants predominantly with the mother's own raw milk results in better neonatal outcomes such as higher body length and head circumference at discharge [69]. In addition, a prospective cohort study conducted with VLBW infants fed exclusively with HM demonstrated an association between the early use of the mother's own raw milk and continued breastfeeding at discharge (OR 2.92; 95% CI [1.94–4.40]), and after 6 months (OR 2.70; 95% CI [1.21–6.03]) [70].

In view of all the arguments presented, the concentrated HM produced in this study is an innovative, viable, and less onerous proposal after initial investment, which can provide many benefits to VLBW infants as well as national and international health systems. Finally, the last aim of this study was to evaluate the stability of the concentrated HM samples stored for 3 and 6 months. After 3 months of storage, comparing samples of HMCI and HM3m, the Ca and P contents increased and the levels of energy, total lipids, and Cu were reduced. However, comparing samples of HMCI and HM6m, a significant increase in the levels of Ca, Mg, K, Zn, and P was observed, despite a slight reduction in levels of energy and total lipids, while the osmolality and other nutrients remained stable, except for Cu. The Cu content presented a different pattern in relation to the rest, since it was the only one that suffered a high reduction after the storage period. Despite significant changes in the nutritional content of concentrated HM stored for 3 and 6 months, we considered clinically relevant only the increase of K and the reduction of Cu in the samples. In view of these results, it is possible to preserve the concentrated HM for 6 months; however, it is necessary to consider the changes in the nutritional content of this product, mainly in the K and Cu elements, which suggest the non-storage of this concentrated product. Currently, target fortification has been tested as a method for adjusting macronutrient and micronutrient content in fortified HM with marketed products that even after fortification may not fully meet the nutritional needs of VLBW infants, unlike the concentrated HM produced in this study [71–72]. Several authors suggest that target fortification is safe and capable of individually optimizing the intake of macronutrients (protein, carbohydrate, and fat) by VLBW infants according to the ESPGHAN 2010 guidelines [73–74]. One limitation of this study was the lack of evaluation of diet composition of the donors during the pre-gestational, gestational, and post -gestational periods.

## 5. Conclusions

The present study confirms the possibility of formulating concentrated HM, generated from the freeze-drying of the milk donated from the HMB, with osmolality and levels of certain macronutrients and micronutrients compatible with the nutritional needs of VLBW infants. It should be noted that the simplified direct lyophilization method for the formulation of our concentrated HM minimizes the risk of contamination due to minimum product handling as well as present low cost after an initial investment and does not characterize ethical bias since the milk was donated. In addition, the HM samples stored for 3 and 6 months were evaluated and their osmolality stability and nutrient content stability were verified, with both increases and reductions of certain nutrients, such as K and Cu. Another issue to be considered is the promising possibility of HM conservation through the freeze-drying process of donated milk. The present authors are committed to future research looking forward to a randomized controlled double blind clinical trial, phase 1 and 2, whose financial support was recently approved by the Brazilian CNPq (National Council for Scientific and Technological Development), in order to evaluate the safety and tolerability, as well as the initial performance of our HM concentrate to be used as a resource for the nutritional support of VLBW infants.

## Supporting information

S1 Fig Boxplot of the descriptive results of osmolality, macronutrients, and micronutrients in samples of types HM baseline, HMCI, HMC3m, and HMC6m. (TIF)

S1 Table Mean and confidence interval 90% for osmolality, macronutrients, and micronutrients expressed per 100 kcal in the samples of types HM baseline, HMCI, HMC3m, and HMC6m. (PDF)

S1 Dataset Database collected available. (XLSX)

S2 Dataset Descriptive statistic of macronutrients. (PDF)

S3 Dataset Descriptive statistic of micronutrients. (PDF)

## References

[1] McNelis K, Fu TT, Poindexter B. Nutrition for the extremely preterm infant. Clin Perinatol. 2017 6;44(2):395–406. 10.1016/j.clp.2017.01.012 28477668

[2] Harding JE, Cormack BE, Alexander T, Alsweiler JM, Bloomfield FH. Advances in nutrition of the newborn infant. Lancet. 2017 4 22;389(10079):1660–1668. 10.1016/S0140-6736(17)30552-4 28443560

[3] Raiten DJ, Steiber AL, Hand RK. Executive summary: evaluation of the evidence to support practice guidelines for nutritional care of preterm infants-the Pre-B Project. Am J Clin Nutr. 2016 2;103(2):599S–605S. 10.3945/ajcn.115.124222 26791179 PMC6459075

[4] Kumar RK, Singhal A, Vaidya U, Banerjee S, Anwar F, Rao S. Optimizing nutrition in preterm low birth weight infants-consensus summary. Front Nutr. 2017 5 26;4:20 10.3389/fnut.2017.00020 28603716 PMC5445116

[5] Stephens BE, Walden RV, Gargus RA, Tucker R, McKinley L, Mance M, et al First-week protein and energy intakes are associated with 18-month developmental outcomes in extremely low birth weight infants. Pediatrics. 2009 5;123(5):1337–43. 10.1542/peds.2008-0211 19403500

[6] Hiltunen H, Löyttyniemi E, Isolauri E, Rautava S. Early nutrition and growth until the corrected age of 2 years in extremely preterm infants. Neonatology. 2018;113(2):100–107. 10.1159/000480633 29131014

[7] Andreas NJ, Kampmann B, Mehring Le-Doare K. Human breast milk: A review on its composition and bioactivity. Early Hum Dev. 2015 11;91(11):629–35. 10.1016/j.earlhumdev.2015.08.013 26375355

[8] Walker A. Breast milk as the gold standard for protective nutrients. J Pediatr. 2010 2;156(2 Suppl):S3–7. 10.1016/j.jpeds.2009.11.021 20105662

[9] Sammallahti S, Kajantie E, Matinolli HM, Pyhälä R, Lahti J, Heinonen K, et al Nutrition after preterm birth and adult neurocognitive outcomes. PLoS One. 2017 9 28;12(9):e0185632 10.1371/journal.pone.0185632 28957424 PMC5619810

[10] Patra K, Hamilton M, Johnson TJ, Greene M, Dabrowski E, Meier PP, et al NICU human milk dose and 20-Month neurodevelopmental outcome in very low birth weight infants. Neonatology. 2017;112(4):330–336. 10.1159/000475834 28768286 PMC5683911

[11] Cortez J, Makker K, Kraemer DF, Neu J, Sharma R, Hudak ML. Maternal milk feedings reduce sepsis, necrotizing enterocolitis and improve outcomes of premature infants. J Perinatol. 2018 1;38(1):71–74. 10.1038/jp.2017.149 29048409

[12] Colaizy TT, Bartick MC, Jegier BJ, Green BD, Reinhold AG, Schaefer AJ, et al Impact of optimized breastfeeding on the costs of necrotizing enterocolitis in extremely low birthweight infants. J Pediatr. 2016 8;175:100–105.e2. 10.1016/j.jpeds.2016.03.040 27131403 PMC5274635

[13] Assad M, Elliott MJ, Abraham JH. Decreased cost and improved feeding tolerance in VLBW infants fed an exclusive human milk diet. J Perinatol. 2016 3;36(3):216–20. 10.1038/jp.2015.168 26562370

[14] Dutta S, Singh B, Chessell L, Wilson J, Janes M, McDonald K, et al Guidelines for feeding very low birth weight infants. Nutrients. 2015 1 8;7(1):423–42. 10.3390/nu7010423 25580815 PMC4303848

[15] Bertino E, Giuliani F, Baricco M, Di Nicola P, Peila C, Vassia C, et al Benefits of donor milk in the feeding of preterm infants. Early Hum Dev. 2013 10;89 Suppl 2:S3–6. 10.1016/j.earlhumdev.2013.07.008 23932110

[16] Valentine CJ, Morrow G, Reisinger A, Dingess KA, Morrow AL, Rogers LK. Lactational stage of pasteurized human donor milk contributes to nutrient limitations for infants. Nutrients. 2017 3 18;9(3). pii: E302 10.3390/nu9030302 28335478 PMC5372965

[17] Colaizy TT, Carlson S, Saftlas AF, Morriss FH Jr. Growth in VLBW infants fed predominantly fortified maternal and donor human milk diets: a retrospective cohort study. BMC Pediatr. 2012 8 17;12:124 10.1186/1471-2431-12-124 22900590 PMC3464178

[18] Morlacchi L, Roggero P, Giannì ML, Bracco B, Porri D, Battiato E, et al Protein use and weight-gain quality in very-low-birth-weight preterm infants fed human milk or formula. Am J Clin Nutr. 2018 2 1;107(2):195–200. 10.1093/ajcn/nqx001 29529139

[19] Bhatia J, Griffin I, Anderson D, Kler N, Domellöf M. Selected macro/micronutrient needs of the routine preterm infant. J Pediatr. 2013 3;162(3 Suppl):S48–55. 10.1016/j.jpeds.2012.11.053 23445848

[20] Terrin G, Berni Canani R, Di Chiara M, Pietravalle A, Aleandri V, Conte F, et al Zinc in Early Life: A Key Element in the fetus and preterm neonate. Nutrients. 2015 12 11;7(12):10427–46. 10.3390/nu7125542 26690476 PMC4690094

[21] Abiramalatha T, Thomas N, Gupta V, Viswanathan A, McGuire W. High versus standard volume enteral feeds to promote growth in preterm or low birth weight infants. Cochrane Database Syst Rev. 2017 9 12;9:CD012413 10.1002/14651858.CD012413.pub2 28898404 PMC6483816

[22] Kreissl A, Zwiauer V, Repa A, Binder C, Haninger N, Jilma B, et al Effect of fortifiers and additional protein on the osmolarity of human milk: is it still safe for the premature infant? J Pediatr Gastroenterol Nutr. 2013 10;57(4):432–7. 10.1097/MPG.0b013e3182a208c7 23857340

[23] Abrams SA, Schanler RJ, Lee ML, Rechtman DJ. Greater mortality and morbidity in extremely preterm infants fed a diet containing cow milk protein products. Breastfeed Med. 2014 Jul-Aug;9(6):281–5. 10.1089/bfm.2014.0024 24867268 PMC4074755

[24] Hair AB, Peluso AM, Hawthorne KM, Perez J, Smith DP, Khan JY, et al Beyond necrotizing enterocolitis prevention: Improving outcomes with an exclusive human milk-based diet. Breastfeed Med. 2016 3;11(2):70–4. 10.1089/bfm.2015.0134 26789484 PMC4782036

[25] Brown JV, Embleton ND, Harding JE, McGuire W. Multi-nutrient fortification of human milk for preterm infants. Cochrane Database Syst Rev. 2016 5 8;(5):CD000343 10.1002/14651858.CD000343.pub3 27155888

[26] Hair AB, Blanco CL, Moreira AG, Hawthorne KM, Lee ML, Rechtman DJ, et al Randomized trial of human milk cream as a supplement to standard fortification of an exclusive human milk-based diet in infants 750–1250 g birth weight. J Pediatr. 2014 11;165(5):915–20. 10.1016/j.jpeds.2014.07.005 25130571

[27] Ganapathy V, Hay JW, Kim JH. Costs of necrotizing enterocolitis and cost-effectiveness of exclusively human milk-based products in feeding extremely premature infants. Breastfeed Med. 2012 2;7(1):29–37. 10.1089/bfm.2011.0002 21718117

[28] Carroll K, Herrmann KR. The cost of using donor human milk in the NICU to achieve exclusively human milk feeding through 32 weeks postmenstrual age. Breastfeed Med. 2013 6;8(3): 286–90. 10.1089/bfm.2012.0068 23323965 PMC3663453

[29] Hair AB, Bergner EM, Lee ML, Moreira AG, Hawthorne KM, Rechtman DJ, et al Premature infants 750–1,250 g birth weight supplemented with a novel human milk-derived cream are discharged sooner. Breastfeed Med. 2016 4;11:133–7. 10.1089/bfm.2015.0166 26982282 PMC4827298

[30] Escuder-Vieco D, Vázquez-Román S, Sánchez-Pallás J, Ureta-Velasco N, Mosqueda-Peña R, Pallás-Alonso CR. Determination of acidity in donor milk. J Hum Lact. 2016 11;32(4):NP73–NP75. 10.1177/0890334415591338 26116636

[31] Grazziotin MC, Grazziotin AL, Vidal NM, Freire MH, da Silva RP. Analysis of the storage methods for raw human milk from mothers with infants admitted to a neonatal intensive care unit, according to Brazilian Regulations. J Hum Lact. 2016 8;32(3):446–54. 10.1177/0890334416647710 27165765

[32] Picaud JC, Buffin R. Human milk-treatment and quality of banked human milk. Clin Perinatol. 2017 3;44(1):95–119. 10.1016/j.clp.2016.11.003 28159212

[33] Haiden N, Ziegler EE. Human milk banking. Ann Nutr Metab. 2016;69 Suppl 2:8–15. 10.1159/000452821 28103607

[34] Peila C, Moro GE, Bertino E, Cavallarin L, Giribaldi M, Giuliani F, et al The Effect of holder pasteurization on nutrients and biologically active components in donor human milk: A review. Nutrients. 2016 8 2;8(8). pii: E477 10.3390/nu8080477 27490567 PMC4997390

[35] Sullivan S, Schanler RJ, Kim JH, Patel AL, Trawöger R, Kiechl-Kohlendorfer U, et al An exclusively human milk-based diet is associated with a lower rate of necrotizing enterocolitis than a diet of human milk and bovine milk-based products. J Pediatr. 2010 4;156(4):562–7. 10.1016/j.jpeds.2009.10.040 20036378

[36] Perrella SL, Hepworth AR, Simmer KN, Geddes DT. Influences of breast milk composition on gastric emptying in preterm infants. J Pediatr Gastroenterol Nutr. 2015 2;60(2):264–71. 10.1097/MPG.0000000000000596 25313848

[37] Thomaz DM, Serafim PO, Palhares DB, Melnikov P, Venhofen L, Vargas MO. Comparison between homologous human milk supplements and a commercial supplement for very low birth weight infants. J Pediatr (Rio J). 2012 Mar-Apr;88(2):119–24. 22406953 10.2223/JPED.2166

[38] Shulhan J, Dicken B, Hartling L, Larsen BM. Current knowledge of necrotizing enterocolitis in preterm infants and the impact of different types of enteral nutrition products. Adv Nutr. 2017 1 17;8(1):80–91. 10.3945/an.116.013193 28096129 PMC5227976

[39] Grance TR, Serafin Pde O, Thomaz DM, Palhares DB. Homologous human milk supplement for very low birth weight preterm infant feeding. Rev Paul Pediatr. 2015 Jan-Mar;33(1):28–33. 10.1016/j.rpped.2014.07.001 25662564 PMC4436953

[40] Rosas R, Sanz MP, Fernández-Calle P, Alcaide MJ, Montes MT, Pastrana N, et al Experimental study showed that adding fortifier and extra-hydrolysed proteins to preterm infant mothers' milk increased osmolality. Acta Paediatr. 2016 12;105(12):e555–e560. 10.1111/apa.13522 27392326

[41] Mohd-Taufek N, Cartwright D, Davies M, Hewavitharana AK, Koorts P, McConachy H, et al The effect of pasteurization on trace elements in donor breast milk. J Perinatol. 2016 10;36(10):897–900. 10.1038/jp.2016.88 27253894

[42] Lozano B, Castellote AI, Montes R, López-Sabater MC. Vitamins, fatty acids, and antioxidant capacity stability during storage of freeze-dried human milk. Int J Food Sci Nutr. 2014 9;65(6):703–7. 10.3109/09637486.2014.917154 24840090

[43] Cortez MV, Soria EA. The effect of freeze-drying on the nutrient, polyphenol, and oxidant levels of breast milk. Breastfeed Med. 2016 12;11:551–554. 10.1089/bfm.2016.0102 27925493

[44] Tudehope D, Fewtrell M, Kashyap S, Udaeta E. Nutritional needs of the micropreterm infant. J Pediatr. 2013 3;162(3 Suppl):S72–80. 10.1016/j.jpeds.2012.11.056 23445852

[45] Agostoni C, Buonocore G, Carnielli VP, De Curtis M, Darmaun D, Decsi T, et al Enteral nutrient supply for preterm infants: commentary from the European Society of Paediatric Gastroenterology, Hepatology and Nutrition Committee on Nutrition. J Pediatr Gastroenterol Nutr. 2010 1;50(1):85–91. 10.1097/MPG.0b013e3181adaee0 19881390

[46] Westin V, Klevebro S, Domellöf M, Vanpée M, Hallberg B, Stoltz Sjöström E. Improved nutrition for extremely preterm infants—A population based observational study. Clin Nutr ESPEN. 2018 2;23:245–251. 10.1016/j.clnesp.2017.09.004 29460807

[47] Schneider J, Fischer Fumeaux CJ, Duerden EG, Guo T, Foong J, Graz MB, et al Nutrient intake in the first two weeks of life and brain growth in preterm neonates. Pediatrics. 2018;141(3):e20172169 10.1542/peds.2017-2169 29440285

[48] Spiegler J, Preuß M, Gebauer C, Bendiks M, Herting E, Göpel W, et al Does breastmilk influence the development of bronchopulmonary Dysplasia? J Pediatr. 2016 2;169:76–80.e4. 10.1016/j.jpeds.2015.10.080 26621048

[49] Lewis ED, Richard C, Larsen BM, Field CJ. The importance of human milk for immunity in preterm infants. Clin Perinatol. 2017 3;44(1):23–47. 10.1016/j.clp.2016.11.008 28159208

[50] Lönnerdal B. Bioactive proteins in human milk-potential benefits for preterm infants. Clin Perinatol. 2017 3;44(1):179–191. 10.1016/j.clp.2016.11.013 28159205

[51] Koletzko B. Should women providing milk to their preterm infants take Docosahexaenoic acid supplements? Clin Perinatol. 2017 3;44(1):85–93. 10.1016/j.clp.2016.11.002 28159211

[52] Senterre T, Abu Zahirah I, Pieltain C, de Halleux V, Rigo J. Electrolyte and mineral homeostasis after optimizing early macronutrient intakes in VLBW infants on parenteral nutrition. J Pediatr Gastroenterol Nutr. 2015 10;61(4):491–8. 10.1097/MPG.0000000000000854 25988555

[53] Körnmann MN, Christmann V, Gradussen CJW, Rodwell L, Gotthardt M, Van Goudoever JB, et al Growth and bone mineralization of very preterm infants at term corrected age in relation to different nutritional intakes in the early postnatal period. Nutrients. 2017 12 2;9(12). pii: E1318 10.3390/nu9121318 29207479 PMC5748768

[54] Harding JE, Wilson J, Brown J. Calcium and phosphorus supplementation of human milk for preterm infants. Cochrane Database Syst Rev. 2017 2 26;2:CD003310 10.1002/14651858.CD003310.pub2 28238222 PMC6464224

[55] Rigo J, Pieltain C, Christmann V, Bonsante F, Moltu SJ, Iacobelli S, et al Serum magnesium levels in preterm infants are higher than adult levels: A systematic literature review and meta-analysis. Nutrients. 2017 10 16;9(10). pii:E1125. 10.3390/nu9101125 29035309 PMC5691741

[56] Terrin G, Berni Canani R, Passariello A, Messina F, Conti MG, Caoci S, et al Zinc supplementation reduces morbidity and mortality in very-low-birth-weight preterm neonates: a hospital-based randomized, placebo-controlled trial in an industrialized country. Am J Clin Nutr. 2013 12;98(6):1468–74. 10.3945/ajcn.112.054478 24025633

[57] Kojima C, Shoji H, Ikeda N, Kitamura T, Hisata K, Shimizu T. Association of zinc and copper with clinical parameters in the preterm newborn. Pediatr Int. 2017 11;59(11):1165–1168. 10.1111/ped.13409 28851072

[58] Watson L, Cartwright D, Jardine LA, Pincus D, Koorts P, Kury S, et al Transient neonatal zinc deficiency in exclusively breastfed preterm infants. J Paediatr Child Health. 2018 3;54(3):319–322. 10.1111/jpc.13780 29143460

[59] Marquardt ML, Done SL, Sandrock M, Berdon WE, Feldman KW. Copper deficiency presenting as metabolic bone disease in extremely low birth weight, short-gut infants. Pediatrics. 2012 9;130(3):e695–8. 10.1542/peds.2011-1295 22869833

[60] Lava SA, Bianchetti MG, Simonetti GD. Salt intake in children and its consequences on blood pressure. Pediatr Nephrol. 2015 9;30(9):1389–96. 10.1007/s00467-014-2931-3 25127918

[61] Isemann B, Mueller EW, Narendran V, Akinbi H. Impact of Early Sodium Supplementation on Hyponatremia and Growth in Premature Infants: A Randomized Controlled Trial. JPEN J Parenter Enteral Nutr. 2016 3;40(3):342–9. 10.1177/0148607114558303 25406227

[62] Koo W, Tice H. Human milk fortifiers do not meet the current recommendation for nutrients in very low birth weight infants. JPEN J Parenter Enteral Nutr. 2018 5;42(4):813–820. 10.1177/0148607117713202 28622483

[63] Einloft PR, Garcia PC, Piva JP, Schneider R, Fiori HH, Fiori RM. Supplemented vs. unsupplemented human milk on bone mineralization in very low birth weight preterm infants: a randomized clinical trial. Osteoporos Int. 2015 9;26(9): 2265–71. 10.1007/s00198-015-3144-8 25971686

[64] Denning TL, Bhatia AM, Kane AF, Patel RM, Denning PW. Pathogenesis of NEC: Role of the innate and adaptive immune response. Semin Perinatol. 2017 2;41(1):15–28. 10.1053/j.semperi.2016.09.014 27940091 PMC5484641

[65] Peila C, Emmerik NE, Giribaldi M, Stahl B, Ruitenberg JE, van Elburg RM, et al Human milk processing: A systematic review of innovative techniques to ensure the safety and quality of donor milk. J Pediatr Gastroenterol Nutr. 2017 3;64(3):353–361. 10.1097/MPG.0000000000001435 27755345

[66] Ahrabi AF, Handa D, Codipilly CN, Shah S, Williams JE, McGuire MA, et al Effects of extended freezer storage on the integrity of human milk. J Pediatr. 2016 10;177:140–143. 10.1016/j.jpeds.2016.06.024 27423174

[67] Sousa SG, Delgadillo I, Saraiva JA. Effect of thermal pasteurisation and high-pressure processing on immunoglobulin content and lysozyme and lactoperoxidase activity in human colostrum. Food Chem. 2014 5 15;151:79–85. 10.1016/j.foodchem.2013.11.024 24423505

[68] Gidrewicz DA, Fenton TR. A systematic review and meta-analysis of the nutrient content of preterm and term breast milk. BMC Pediatr. 2014 8 30;14:216 10.1186/1471-2431-14-216 25174435 PMC4236651

[69] Dritsakou K, Liosis G, Valsami G, Polychronopoulos E, Skouroliakou. Improved outcomes of feeding low birth weight infants with predominantly raw human milk versus donor banked milk and formula. J Matern Fetal Neonatal Med. 2016;29(7):1131–8. 10.3109/14767058.2015.1038232 25909500

[70] Fischer Fumeaux CJ, Denis A, Prudon MB, Plaisant F, Essomo Megnier-Mbo CM, Fernandes L, et al Early use of mother's own raw milk, maternal satisfaction, and breastfeeding continuation in hospitalised neonates: A prospective cohort study. Neonatology. 2018;113(2):131–139. 10.1159/000480535 29186707

[71] Choi A, Fusch G, Rochow N, Fusch C. Target fortification of breast milk: Predicting the final osmolality of the feeds. PLoS One. 2016 2 10;11(2):e0148941 10.1371/journal.pone.0148941 26863130 PMC4749227

[72] de Halleux V, Rigo J. Variability in human milk composition: benefit of individualized fortification in very-low-birth-weight infants. Am J Clin Nutr. 2013 8;98(2):529S–35S. 10.3945/ajcn.112.042689 23824725

[73] Rochow N, Fusch G, Choi A, Chessell L, Elliot L, McDonald K, et al Target fortification of breast milk with fat, protein, and carbohydrates for preterm infants. J Pediatr. 2013 10;163(4):1001–7. 10.1016/j.jpeds.2013.04.052 23769498

[74] Rochow N, Fusch G, Zapanta B, Ali A, Barui S, Fusch C. Target fortification of breast milk: how often should milk analysis be done? Nutrients. 2015 4 1;7(4):2297–310. 10.3390/nu7042297 25835073 PMC4425145

