# Supplementary material for: Development of a human milk concentrate with human milk lyophilizate for feeding very low birth weight preterm infants: A preclinical experimental study
Source: PLoS One. 2019 Feb 20;14(2):e0210999. doi: 10.1371/journal.pone.0210999 (PMC6382113; doi:10.1371/journal.pone.0210999)
Supplement: S1 Table — (PDF) [file pone.0210999.s002.pdf]

S1 Table. Mean and confidence interval 90% for osmolality, macronutrients, and micronutrients expressed per 100 kcal in the samples of types HM baseline, HMCI, HMC3m, and HMC6m.

Dosages (units)	HM baseline	HMCI	HMC3m	HMC6m
Volume (ml)	177.6	125.1	129.9	129.7
Protein (g)	1.59 (1.39-1.80)	1.85 (1.68-2.02)	1.81 (1.62-1.99)	1.91 (1.74-2.07)
Carbohydrate (g)	12.57 (12.29-12.85)	11.48 (11.28-11.68)	11.96 (11.77-12.15)	11.90 (11.71-12.10)
Total lipids (g)	4.60 (4.14-5.05)	5.04 (4.61-5.46)	4.78 (4.36-5.19)	4.79 (4.37-5.20)
Total solids (g)	19.11 (18.56-19.66)	18.48 (17.96-18.98)	18.81 (18.26-19.35)	18.85 (18.32-19.36)
True Protein (g)	1.33 (1.16-1.50)	1.50 (1.36-1.64)	1.47 (1.31-1.62)	1.54 (1.41-1.67)
Osmolality (mOsm/Kg H_20)	289.48 (279.17-396.25)	452.12 (437.99-559.85)	456.16 (442.79-564.36)	458.14 (444.99-458.14)
Calcium (mg)	41.27 (39.30-43.24)	45.70 (43.56-47.81)	50.23 (48.31-52.15)	52.69 (50.85-54,54)
Magnesium (mg)	4.62 (4.38-4.85)	5.13 (4.89-5.36)	5.55 (5.51-5.58)	5.87 (5.84-5.90)
Sodium (mg)	23.98 (19.87-28.09)	27.84 (22.84-32.83)	31.81 (26.81-36.80)	30.24 (25.90-35.58)
Potassium (mg)	106.81 (100.59-113.01)	126.83 (118.87-134.79)	131.85 (125.01-138.68)	149.43 (142.26-156.60)
Copper (µg)	59.82 (53.69-65.93)	60.42 (54.51-66.33)	53.47 (48.82-58.10)	50.95 (46.74-55.14)
Zinc (mg)	0.26 (0.21-0.32)	0.25 (0.22-0.29)	0.29 (0.24-0.33)	0.33 (0.28-0.37)
Phosphorus (mg)	25.9 (23.43-28.53)	23.10 (21.34-24.87)	26.45 (24.14-28.75)	26.59 (24.33-28.84)

HM baseline: Human milk baseline; HMCI: HM concentrated for immediate analysis; HMC3m: HM concentrate for analysis after 3 months of storage; HMC6m: HM concentrate for analysis after 6 months of storage; Mean (confidence interval 90%).
