# Supplementary material for: Development of a human milk concentrate with human milk lyophilizate for feeding very low birth weight preterm infants: A preclinical experimental study
Source: PLoS One. 2019 Feb 20;14(2):e0210999. doi: 10.1371/journal.pone.0210999 (PMC6382113; doi:10.1371/journal.pone.0210999)
Supplement: S2 Dataset — (PDF) [file pone.0210999.s004.pdf]

The SAS System

The MEANS Procedure

Time	N Obs	Variable	Mean	Std Dev	Minimum	Lower Quartile	Median	Upper Quartile	Maximum
HM baseline	50	Total lipids	2.59	1.08	0.80	1.70	2.50	3.30	5.00
		Protein	0.90	0.49	0.30	0.60	0.80	1.00	2.80
		Carbohydrate	7.08	0.67	5.10	6.60	7.20	7.40	8.70
		Total solids	10.76	1.31	8.20	9.80	10.75	11.90	13.70
		Energy	56.30	10.51	37.00	48.00	55.00	65.00	80.00
		True protein	0.75	0.40	0.20	0.50	0.70	0.90	2.30
		Osmolality	289.48	43.64	182.00	275.00	292.00	299.00	477.00
HMCI	50	Total lipids	4.03	1.44	1.30	3.20	4.00	4.80	7.50
		Protein	1.48	0.58	0.50	1.10	1.40	1.70	3.80
		Carbohydrate	9.18	0.68	7.50	8.70	9.30	9.70	10.10
		Total solids	14.77	1.72	11.30	13.70	14.65	15.90	18.40
		Energy	79.96	13.75	56.00	70.00	78.50	89.00	109.00
		True protein	1.20	0.48	0.40	0.90	1.10	1.40	3.10
		Osmolality	452.12	59.79	338.00	417.00	467.50	493.00	564.00
HMC3m	50	Total lipids	3.68	1.34	1.30	2.80	3.70	4.50	6.80
		Protein	1.39	0.61	0.40	1.00	1.30	1.70	3.70
		Carbohydrate	9.21	0.63	7.80	8.80	9.35	9.70	10.10
		Total solids	14.48	1.78	10.70	13.30	14.45	15.70	18.30
		Energy	76.98	13.91	52.00	67.00	76.50	86.00	109.00
		True protein	1.13	0.49	0.20	0.90	1.05	1.40	3.00
		Osmolality	456.16	56.58	330.00	427.00	472.50	496.00	557.00
HMC6m	50	Total lipids	3.69	1.35	1.20	2.80	3.65	4.40	7.10
		Protein	1.47	0.54	0.70	1.10	1.40	1.60	3.80
		Carbohydrate	9.18	0.64	7.70	8.80	9.35	9.70	10.20
		Total solids	14.53	1.71	11.20	13.30	14.45	15.50	18.80
		Energy	77.30	13.78	54.00	68.00	75.50	84.00	113.00
		True protein	1.19	0.43	0.60	0.90	1.10	1.30	3.10
		Osmolality	458.14	55.67	338.00	430.00	473.00	497.00	559.00
