# Supplementary material for: Development of a human milk concentrate with human milk lyophilizate for feeding very low birth weight preterm infants: A preclinical experimental study
Source: PLoS One. 2019 Feb 20;14(2):e0210999. doi: 10.1371/journal.pone.0210999 (PMC6382113; doi:10.1371/journal.pone.0210999)
Supplement: S3 Dataset — (PDF) [file pone.0210999.s005.pdf]

The SAS System

The MEANS Procedure

Time	N Obs	Variable	Mean	Std Dev	Minimum	Lower Quartile	Median	Upper Quartile	Maximum
HM baseline	50	Sodium	135.04	98.03	22.00	70.00	107.00	146.00	464.00
		Potassium	601.38	147.98	336.10	512.00	578.60	683.00	1066.50
		Calcium	23.24	4.70	13.00	20.20	22.30	27.40	31.20
		Magnesium	2.14	0.46	1.34	1.80	2.04	2.42	3.52
		Phosphorus	14.63	6.09	6.50	10.90	13.15	16.70	45.40
		Zinc	149.10	128.15	22.70	70.00	105.00	191.00	650.00
		Copper	33.37	14.60	14.00	22.60	30.10	41.80	84.60
HMCI	50	Sodium	222.52	169.03	60.00	116.00	166.00	244.00	792.00
		Potassium	1013.85	269.45	530.70	828.80	991.05	1155.20	1913.30
		Calcium	36.52	7.18	20.60	31.80	37.00	41.20	53.20
		Magnesium	3.38	0.65	1.74	2.88	3.24	3.94	5.36
		Phosphorus	18.47	5.97	8.00	14.15	17.35	22.50	34.10
		Zinc	203.89	126.20	34.60	110.00	182.75	256.00	570.00
		Copper	48.30	19.99	21.00	34.20	43.80	58.20	125.00
HMC3m	50	Sodium	244.87	162.80	76.00	138.00	198.00	280.00	700.00
		Potassium	1015.04	222.82	520.00	855.20	1004.60	1131.50	1671.20
		Calcium	38.67	6.25	24.20	36.20	38.20	42.80	50.76
		Magnesium	3.52	0.83	2.32	2.88	3.37	4.04	6.38
		Phosphorus	20.36	7.52	9.95	15.20	18.33	24.95	41.75
		Zinc	224.46	152.50	53.50	108.50	198.50	282.00	715.00
		Copper	41.16	15.12	19.60	29.00	41.10	48.40	93.60
HMC6m	50	Sodium	233.19	141.53	76.00	132.00	185.00	280.00	710.00
		Potassium	1152.31	234.18	730.30	967.90	1086.85	1267.80	1783.10
		Calcium	40.63	6.02	26.00	38.40	41.00	45.39	55.45
		Magnesium	3.73	0.90	2.34	3.08	3.55	4.32	6.14
		Phosphorus	20.50	7.35	9.55	13.85	19.70	24.90	42.10
		Zinc	251.70	158.76	48.00	129.50	212.25	328.00	715.00
		Copper	39.28	13.71	15.60	27.60	38.80	47.20	72.00
